# Selective segregation of DNA strands persists in long label retaining mammary cells during pregnancy

**DOI:** 10.1186/bcr2188

**Published:** 2008-10-24

**Authors:** Brian W Booth, Corinne A Boulanger, Gilbert H Smith

**Affiliations:** 1 Mammary Biology and Tumorigenesis Laboratory, National Cancer Institute, Convent Drive, National Institute of Health, Bethesda, MD 20892, USA

## Abstract

**Introduction:**

During pregnancy the mammary epithelial compartment undergoes extreme proliferation and differentiation, facilitated by stem/progenitor cells. Mouse mammary epithelium in nonpregnant mice contains long label-retaining epithelial cells (LREC) that divide asymmetrically and retain their template DNA strands. The role of LREC during alveogenesis has not been determined.

**Methods:**

We performed immunohistochemistry and autoradiography on murine mammary glands that had been labeled with 5-bromodeoxyuridine (5BrdU) during allometric ductal growth to investigate the co-expression of DNA label retention and estrogen receptor-α or progesterone receptor during pregnancy. A second DNA label ([^3^H]-thymidine) was administered during pregnancy to identify label-retaining cells (LRC), which subsequently enter the cell cycle. Use of this methodology allowed us to investigate the co-localization of 5BrdU with smooth muscle actin, CD31, cytokeratin, and desmin in periductal or peri-acinar LRC in mammary tissue from pregnant mice subsequent to a long chase period in order to identify LRC.

**Results:**

Estrogen receptor-α positive and progesterone receptor positive cells represented approximately 30% to 40% of the LREC, which is under 1.0% of the epithelial subpopulation. Pregnancy altered the percentage of LREC expressing estrogen receptor-α. LRC situated in periductal or peri-acinar positions throughout the gland do not express epithelial, endothelial, or myoepithelial markers, and these undefined LRCs persist throughout pregnancy. Additionally, new cycling LREC ([^3^H]-thymidine retaining) appear during alveologenesis, and LRC found in other tissue types (for example, endothelium and nerve) within the mammary fat pad become double labeled during pregnancy, which indicates that they may also divide asymmetrically.

**Conclusions:**

Our findings support the premise that there is a subpopulation of LREC in the mouse mammary gland that persists during alveologenesis. These cells react to hormonal cues during pregnancy and enter the cell cycle while continuing to retain, selectively, their original template DNA. In addition, nonepithelial LRC are found in periductal or peri-acinar positions. These LRC also enter the cell cycle during pregnancy. During alveologenesis, newly created label-retaining ([^3^H]-thymidine) epithelial cells appear within the expanding alveoli and continue to cycle and retain their original template DNA ([^3^H]-thymidine) strands, as determined by a second pulse of 5BrdU.

## Introduction

In 1975, Cairns [1] postulated that dividing adult somatic stem cells protected themselves from mutation and cancer risk by segregating their template DNA strands. This property of selective DNA segregation was shown to occur both *in vivo *and *in vitro *in a variety of cell and tissue types [2-8]. In the mouse mammary gland, label-retaining epithelial cells (LREC) in the ducts divide asymmetrically and retain their template DNA strands [6]. In addition, more than 80% of these LREC remain in the cell cycle dividing actively (as indicated by their incorporation of a second DNA label after a 48-hour pulse), and subsequently – upon chase – bestow the newly labeled DNA strands upon their progeny during asymmetric cell divisions [6]. Ductal morphogenesis occurs between weeks 3 and 10 of age in the mouse, during which the mammary gland is rapidly proliferating and differentiating [9]. Mammary epithelial stem cells contribute to this development of the mammary gland by asymmetric division, generating both transit-amplifying and progenitor cells that subsequently divide by symmetric division, resulting in mammary stem cell expansion. It is commonly believed that in the mature mouse the mammary epithelium exists in a state of relative proliferative quiescence, with the exception of short bursts during hormonal stimulation manifesting as the estrus cycle, until the onset of pregnancy [10,11]. Recent studies have indicated that considerable cellular turnover occurs in mammary epithelium [12-14]. Tissue homeostasis is maintained during this period by stem/progenitor cells positioned throughout the mammary ductal system.

Undifferentiated mammary stem/progenitor cells are found microscopically, based on morphologic characteristics in rats and mice, in suprabasal positions between the luminal and myoepithelial cell layers [15]. The presence of these cells with similar characteristics has also been confirmed in human mammary glands [16,17]. These morphologically undifferentiated epithelial cells persist in immortalized, hyperplastic mammary tissue but are absent from growth senescent populations [18]. These studies provided additional evidence for mammary stem cells that reside among the epithelial components of the gland (for review [19]).

The importance of estrogen-mediated and progesterone-mediated proliferation in normal mammary growth and development is well documented [20]. We previously reported that a subpopulation of LREC in the murine mammary gland is positive for estrogen receptor (ER)-α and/or progesterone receptor (PR), and that the numbers of these cells were altered by administration of various hormones (estrogen, progesterone, and prolactin), either alone or in combination [21]. We also communicated our findings that a number of nonepithelial nuclear label-retaining cells (LRC) reside in periductal or basal positions throughout the mammary gland. This report characterizes these cells further by following them through pregnancy as well as assessing LREC for ER-α and PR expression during early pregnancy. Furthermore, we examined developing and expanding alveoli for the appearance of newly formed LREC that are capable of cycling asymmetrically while retaining their original [^3^H]-thymidine DNA label.

## Materials and methods

### Experimental plan

We conducted two separate procedures designed to label the DNA of proliferating cells during the period of ductal elongation and development. In the first experiment mammary glands were labeled by administration of 5-bromodeoxyuridine (5BrdU; 1 mg) by intraperitoneal injection for 14 days starting on the first day of week 4 of postnatal life. The label was chased from the glands over a 6-week period after the final injection to allow completion of ductal development. In the second procedure, 5BrdU was administered for 2 consecutives days, once each day, by intraperitoneal injection for 7 weeks beginning on week 4 of postnatal life and ending on week 11. No label was administered for the next 3 weeks. This constitutes the minimum chase period for the last 5BrdU injection of 3 weeks, the maximum chase (for the first inoculum) being 10 weeks. In the second experiment Nu/Nu hosts were used and WAP-Cre/Rosa26R parous fragments were placed into cleared number 4 and 9 fat pads at age 3 weeks (2 weeks before 5BrdU injections were started).

After the respective chase periods, the females were placed with males and timed pregnancies were initiated by observing the females for vaginal plugs each morning after an evening with a male mouse. The actual interval between the final 5BrdU injection and ensuing pregnancy varied from 14 weeks to 17 weeks for the first experiment and from 5 weeks to 9 weeks for the second. The females were divided into four groups, depending on their stage in pregnancy. (Not all mice were actually pregnant when they received the second DNA label, although they were found with vaginal plugs; these mice served as nonpregnant controls). Tissues from at least four pregnant females were collected in each group.

Group 1 received an intraperitoneal injection of [^3^H]-thymidine (50 μCu) on day 3 of pregnancy, and the mice were killed 2 hours after the injection. Group 2 received an intraperitoneal injection of [^3^H]-thymidine (50 μCu) on day 4 of pregnancy and the mice were killed 2 hours after the injection. Group 3 received an intraperitoneal injection of [^3^H]-thymidine (50 μCu) on day 6 of pregnancy and the mice were killed 2 hours after the injection. Group 4 received an intraperitoneal injection of [^3^H]-thymidine (50 μCu) on day 4 of pregnancy, and intraperitoneal injections of 5BrdU on days 11 and 12 of pregnancy. The mice of group 4 were killed 2 hours after the last 5BrdU injection on day 12 of pregnancy. Group 4 was intended to define long label retaining ([^3^H]-thymidine-positive) alveolar epithelial cells that traverse the cell cycle while retaining their original [^3^H]-thymidine label. Based on the previous elegant studies conducted by Traurig [22,23] and Bresciani [11,24] to elucidate the mammary alveolar epithelial cell cycle duration in pregnant mice, we estimate that at least five to eight mammary epithelial cell cycles were completed in the mammary epithelium of the pregnant mice during the 8-day chase (days 4 to 13) after the [^3^H]-thymidine pulse [11].

The protocols and procedures used to perform the experiments upon the animals were reviewed and approved by the Animal Care and Use Committee at the National Cancer Institute at Frederick (MD, USA). Housing and care during the experimental period conformed to the guidelines provided by the US National Institutes of Health.

### Autoradiography and immunochemistry

For autoradiography, 5 to 6 μm sections were cut, placed upon slides, dewaxed, rehydrated through ethanol, and subsequently dipped in Kodak NTB-2 liquid emulsion (Eastman Kodak Company, Rochester, NY, USA) diluted 1:1 with distilled water. After drying, the slides were stored in lightproof slide boxes at constant humidity and temperature for 20 days. After exposure, the slides were developed in Kodak D-19, washed in distilled water, and fixed in Kodak rapid fixer diluted 1:1 with distilled water. After staining and mounting, the slides were observed and evaluated for autoradiographic grains and for immunostaining. Images were recorded using Kodak digital microscopy documentation system 290 and edited using Adobe Photoshop 7.0 (Adobe, San Jose, CA, USA).

All autoradiographic exposures were performed after immunohistochemistry, the sections were deparaffinized and rehydrated, and the endogenous peroxidase was inactivated with 1% hydrogen peroxide in methanol for 30 minutes. Antibodies used were anti-PR 1:75 (clone A009B; Dako USA, Carpinteria, CA, USA), anti-ER-α 1:50 (clone MC-20; Santa Cruz Biotech, Santa Cruz, CA, USA), anti-5BrdU 1:50 (Dako), anti-smooth muscle actin (SMA) 1:150 (Sigma, St. Loius, MO), anti-desmin 1:100 (The Binding Site, San Diego, CA), and anti-CD31 1:200 (PECAM-1; Santa Cruz). Antigen retrieval was accomplished in accordance with the directions of the manufacturers. Negative tissue controls were included in all immunohistochemical analyses. Vector Blue was used as the chromagen for anti-5BdrU except for slides that received autoradiographic exposure, when diaminobenzidine was used. Diaminobenzidine was used for anti-ER-α, anti-PR, anti-SMA, and anti-desmin antibodies. Nova Red was used with anti-CD31 antibody.

Determination of autoradiographic grain counts in epithelial cells was done by counting the grains over at least 100 LRCs in sections from each of the four mammary glands taken from each experimental mouse for a total of 16 mammary glands per experimental group. Determination of antigen expression counts was done using similar methods. At least 500 labeled cells were counted in each of these sections. At least 3,000 nuclei were examined in each slide. Examination of autoradiographic slides from these tissues that were stained for PR and ER-α revealed similar numbers of autoradiographic grains over LREC nuclei.

### Statistical analyses

The GraphPad Prism software package (GraphPad Software, Inc., La Jolla, CA, USA) was used for all statistical analyses. Data were considered significant at *P *< 0.05. Representative data are presented as mean ± standard deviation.

## Results

Several types of long label (5BrdU) retaining cells were observed after the chase period and before pregnancy. These included epithelial and myoepithelial cells, endothelial cells, and cells within the muscle and nerves of the mammary gland. We reported previously that a number of LRCs in the mammary gland reside in periductal (outside the basement membrane along a duct but not stromal fibroblasts) or peri-acinar (outside the basement membrane surrounding a developing acinar structure but not stromal fibroblasts) positions throughout the murine mammary gland [21]. Additionally, we have observed LRECs in basal epithelial positions defined as residing between luminal epithelial cells and myoepithelial cells.

**Figure 1:**
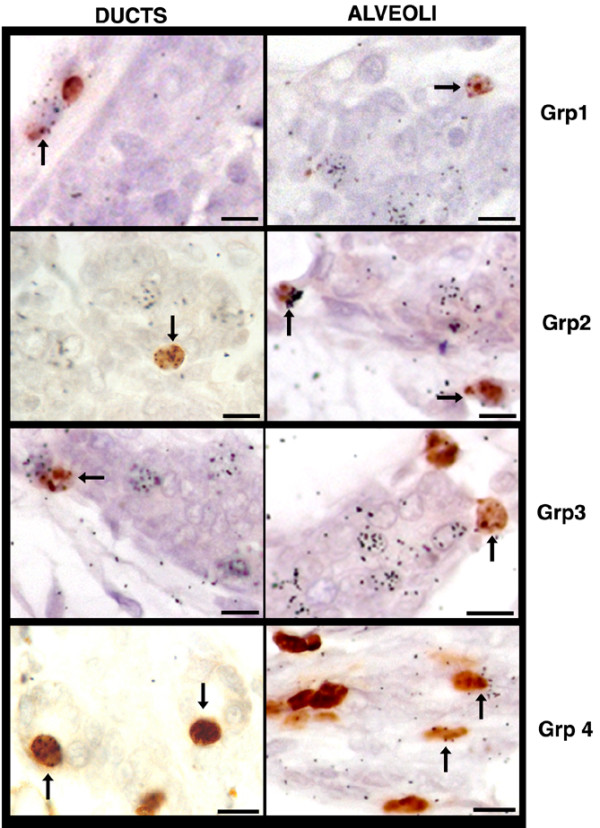
LRC are in cycle during pregnancy. The nuclear label [^3^H]-thymidine is detected by autoradiography in sections of mammary tissue from pregnant mice that received 5BrdU for 2 weeks during weeks 4 and 5 of life (group 1 is day 3 of pregnancy, group 2 is day 4 of pregnancy, group 3 is day 6 of pregnancy, and group 4 is day 12 of pregnancy). LRC in periductal and basal positions are evident in all time points evaluated. Arrows indicate double positive cells. Scale bars = 10 μm. 5BrdU, 5-bromodeoxyuridine; LRC, label-retaining cell.

In order to characterize these cells further, we collected mammary glands from pregnant females that had received 5BrdU constantly for 2 weeks during the allometric growth phase of the mammary gland. These animals were divided into four experimental groups (four animals/group and four glands from each animal were examined), as outlined in the Materials and methods section (above). Each group received a second nuclear label, namely [^3^H]-thymidine, at different time points throughout pregnancy: Group 1 at day 3, Group 2 at day 4, and Group 3 at day 6; the mice in Group 4 received [^3^H]-thymidine at day 4 and also received additional 5BrdU at days 11 and 12, and were killed 2 hours later on pregnancy day 12. Because the onset of pregnancy was not accomplished with synchronicity, the chase period between the last injection of 5BrdU and the [^3^H]-thymidine injection received during pregnancy ranged from 45 to 100 days. We found LRCs (5BrdU-positive cells) in periductal locations and in peri-acinar positions within developing alveoli in all experimental groups (Figure 1).

We next determined whether the periductal LRC and/or the peri-acinar LRC were cycling during pregnancy. The mice in groups 1, 2, and 3 received an injection of [^3^H]-thymidine 1 to 2 hours before death, whereas the mice in group 4 received the [^3^H]-thymidine injection 7 to 8 days before death. Any cells that took up the [^3^H]-thymidine in groups 1 to 3 are considered to be cycling (in S-phase). A subpopulation of the 5BrdU-retaining LRC in groups 1 to 3 was found to be in cycle (Figure 1, arrows). In the pregnant groups, the chase period for 5BrdU exceeded 60 days after the last injection in all cases. In some animals the chase period was greater than 80 days. Therefore, these epithelial cells had maintained their original 5BrdU-labeled DNA for a very long period after incorporation and were in cycle when the pulse of [^3^H]-thymidine was given 1 hour before the mouse was killed. The purpose of group 4, which received [^3^H]-thymidine on day 4 of pregnancy, was to determine whether any of the [^3^H]-thymidine-labeled alveolar cells would still retain this label after an 8-day chase. The mice in group 4 received additional 5BrdU on days 11 and 12 of pregnancy to determine whether any [^3^H]-thymidine-retaining alveolar cells were still traversing the cell cycle. We know that the S-phase of mouse mammary epithelial cells in alveoli of pregnant or hormone-treated mice varies between 11 and 14 hours, based on work conducted by Bresciani [11,24] and Traurig and Morgan [10,22,23]. Thus, if these cells remained in cycle and divided with semiconservative (symmetric) DNA kinetics, then the [^3^H]-thymidine would have been dispersed evenly among the resulting daughters and would be undetectable by autoradiography. Therefore, [^3^H]-thymidine-retaining cells remaining on day 12 of pregnancy were either out of cycle during this period or were in cycle and selectively retained their originally labeled DNA strands. 5BrdU-labeled cells in group 4 numbered about 18% of the total alveolar population. This number compares very well with the observations of Traurig [22] in the alveoli (12%) of 12-day pregnant mice given [^3^H]-thymidine. [^3^H]-thymidine-retaining cells were found among the epithelial alveolar cells on day 13 (7.3%). About 6.1% of the approximately 18% 5BrdU-labeled alveolar cells (76 double labeled of 1,250 5BrdU-positive cells) were also positive for [^3^H]-thymidine (76 double labeled of 283 total [^3^H]-thymidine positive), demonstrating that the tritium-retaining cells were in cycle when 5BrdU was given at day 11 and/or day 12 of pregnancy. We conclude that alveolar LREC arise in alveoli during early pregnancy and function as alveolar cell precursors, which continue to cycle asymmetrically and retain their originally [^3^H]-thymidine labeled DNA during alveolar expansion.

**Figure 2:**
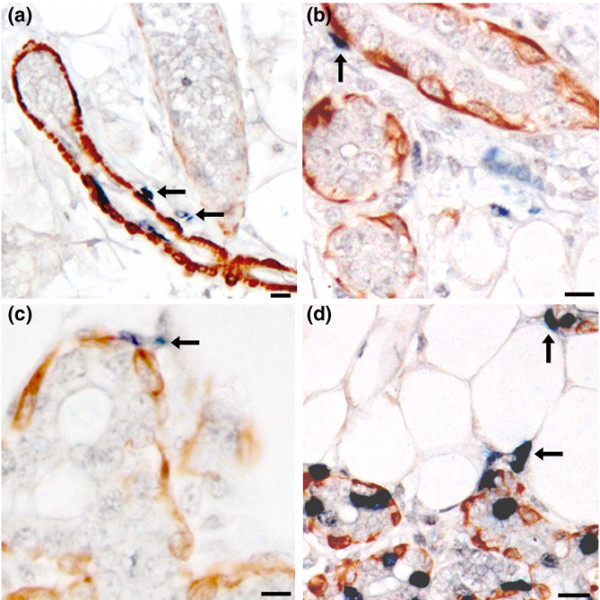
Periductal LRC are not myoepithelial cells. Sections from all experimental groups were stained for the myoepithelial marker SMA (brown). LRC (blue, 5BrdU) in periductal or peri-acinar positions are not SMA positive (arrows). **(a) **Group 1. **(b) **Group 2 cells are endothelial except for arrows. **(c) **Group3. **(d) **Group 4. Scale bars = 15 μm. 5BrdU, 5-bromodeoxyuridine; LRC, label-retaining cell; SMC, smooth muscle actin.

**Figure 3:**
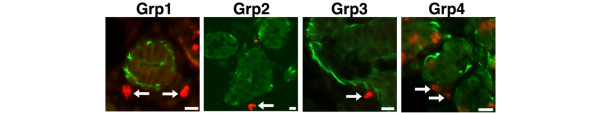
Periductal LRC are not epithelial cells. Sections from all experimental groups were stained for the epithelial markers cytokeratins (green). LRC (red, 5BrdU) in periductal or peri-acinar positions are not cytokertin positive (arrows). Scale bars = 10 μm. 5BrdU, 5-bromodeoxyuridine; LRC, label-retaining cell.

LRC that express the myoepithelial marker SMA were found in all experimental groups. These myoepithelial LRC were found along ducts and developing acinar structures in early pregnancy (group 1; Figure 2a), in later pregnancy (groups 2 and 3; Figures 2b,c) and in mid-pregnancy (group 4; Figure 2d). In late pregnant animals the majority of myoepithelial LRC were located in acini (Figure 2d). In all groups LRC were observed both inside the basement membrane and outside the basement membrane (Figure 2, arrows). In no instance did we find any LRC outside the basement membrane that expressed SMA. Similar results were obtained when we stained for an additional mesenchymal marker, namely desmin (results not shown). Tissue sections from each group were also stained for the epithelial markers cytokeratins. The LRC found in periductal or peri-acinar positions in all experimental groups were not positive for cytokeratin (Figure 3, arrows). These findings indicate that this subset of periductal or peri-acinar LRC do not express either myoepithelial and mesenchymal markers (SMA or desmin) or an epithelial marker (for instance, cytokeratin), leading us to conclude that these cells are undefined cycling cells.

**Figure 4:**
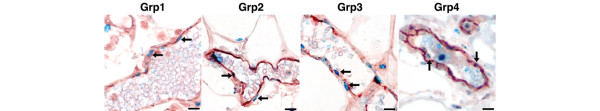
LRC in blood vessels are CD31 positive. Sections from all experimental groups were stained for the endothelial marker CD31 (brown). LRC (blue, 5BrdU) in blood vessels are also CD31 positive. Scale bars = 10 μm. 5BrdU, 5-bromodeoxyuridine; LRC, label-retaining cell.

**Figure 5:**
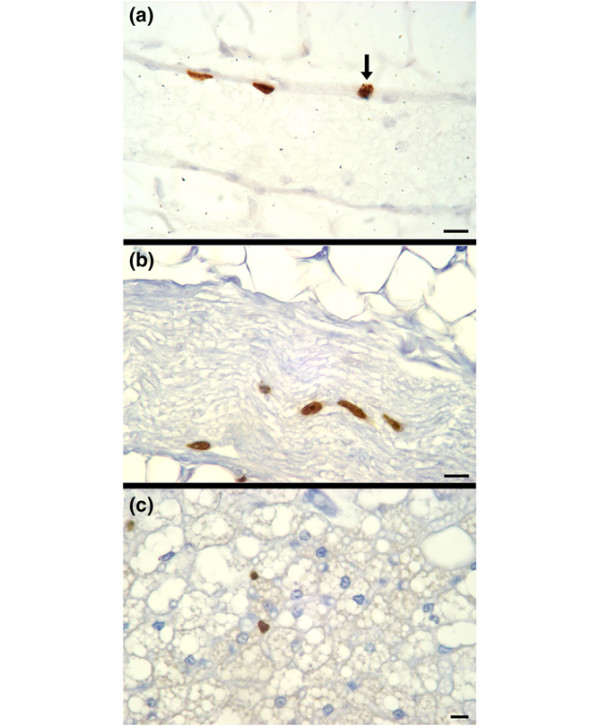
LRC in nonepithelial mammary cells. **(a) **Endothelial cells that line a blood vessel are BrdU positive (brown), and a fraction of those are in cycle, as determined by [^3^H]-thymidine incorporation (arrow). **(b) **LRC exist in a mammary nerve (brown, BrdU positive). **(c) **LRC in fatty stroma (brown, BrdU positive). Scale bars = 10 μm. 5BrdU, 5-bromodeoxyuridine; LRC, label-retaining cell.

To characterize the LRCs further, we determined whether any expressed the endothelial marker CD31 (also known as PECAM-1 [platelet/endothelial cell adhesion molecule 1]). CD31 is expressed on endothelial cells, monocytes, neutrophils, platelets, and some T cells [25]. In none of the groups did we find any epithelial LRC that expressed CD31 or any periductal or peri-acinar CD31^+ ^LRC (results not shown). We found numerous endothelial cells in all experimental groups that were labeled with 5BrdU, after long chase periods, which indicates that some endothelial cells are long-label-retaining (Figure 4). We found that some label-retaining endothelial cells were also [^3^H]-thymidine labeled in pregnant females of groups 1 to 3 that received a pulse, 1 hour before they were killed. Therefore, some long label (5BrdU)-retaining endothelial cells also entered the cell cycle and became double labeled during early pregnancy, presumably corresponding with increased angiogenesis. All LRC lining the blood vessels were CD31 positive, and some were double labeled by the second marker of DNA synthesis, [^3^H]-thymidine (Figure 5a). Additional cell types within the mammary glands also incorporated 5BrdU-forming LRC. Cells within mammary nerves and adipocytes in the fatty stroma retained 5BrdU (Figure 5 panels b and c, respectively) and can be classified as LRC. The formation of LRC is not solely a property of epithelial cells but of all cell types (epithelial, myoepithelial, endothelial, stromal, and neural) that develop within the mammary gland.

**Figure 6:**
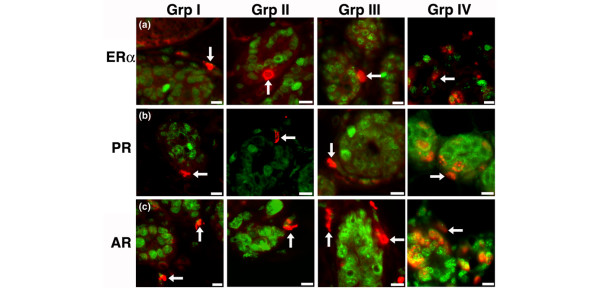
Periductal LRC do not have nuclear hormone receptors. Sections from all experimental groups were stained for the nuclear hormone receptors **(a) **ER-α, **(b) **PR, or **(c) **AR (green). Arrows indicate that periductal LRC (red, 5BrdU) are not double positive for the receptors. Scale bars = 10 μm. AR, adrogen receptor; 5BrdU, 5-bromodeoxyuridine; ER, estrogen receptor; LRC, label-retaining cell; PR, progesterone receptor.

We did not find any instances of periductal or basal LRC (5BrdU positive) that were ER-α positive (Figure 6a) or PR positive (Figure 6b) at any stage during pregnancy. The same was true for androgen receptor expression; specifically, no periductal or basally located LRC were positive for androgen receptor (Figure 6c). This indicates that the periductal LRC do not express any of the three nuclear hormone receptors investigated.

In our previous report [21] we indicated that a number of LREC are ER-α positive and/or PR positive, and these numbers change in response to hormonal stimulation [21]. We next investigated whether the hormonal fluctuations that occur during pregnancy affect the numbers of ER-α-positive and PR-positive LREC. We found a significant decrease in the number of LREC that were also PR positive between group 1 (38.6 ± 8.5%) and group 2 (28.4 ± 6.6; *P *= 0.0446). We also noted a decreasing trend between groups 1 and 3 (29.9 ± 3.0%), although this was not quite statistically significant (*P *= 0.2; Table 1). The decrease in the percentage of PR-positive LREC in group 4 was statistically significant compared with all other experimental groups. In all experimental groups we noted an increase in the percentage of LREC (as defined by 5BrdU retention in groups 1 to 3 and [^3^H]-thymidine retention in group 4) within the PR-positive population when compared with nonpregnant control animals (Table 2). No significant changes were recorded in the ratio of ER-α within the LREC population between groups 1, 2, or 3. These results match our previous findings when we examined LREC after nulliparous animals had received injections of estrogen, progesterone, and prolactin for 5 days [21]. In experimental groups 1 to 3 the percentage of LREC/PR-positive cells within the PR-positive population remained constant but the percentage of PR-positive cells within the entire epithelial population decreased as pregnancy progressed. This suggests that LREC/PR-positive alveolar cells are distinct from the rest of the PR-positive cell population. In group 4 the total number of steroid receptor expressing cells in the entire mammary epithelium decreased by nearly threefold (Table 2). As mentioned above, [^3^H]-thymidine retention defines the long label retaining cells in group 4, and 5BrdU incorporation defines the cells traversing the cell cycle when this label was administered on days 11 and 12 of pregnancy. Any cell that retained [^3^H]-thymidine (defined as autoradiographic detection of at least four grains) was considered label retaining in this analysis. In group 4, nearly one out of every five ER-α-positive cells or PR-positive cells were also [^3^H]-thymidine retaining (Table 2). This indicates that a proportion of the steroid receptor expressing cells are LREC. The observation that the proportion of LREC also positive for these steroid receptors remains unchanged despite the diminished percentage of total nuclear steroid receptor expressing cells supports the interpretation that the steroid receptor positive LREC are distinct from the main steroid receptor positive population.

**Table 1:** Percentages of receptor positive cells within the LREC population

Time point	% ER-α positive^a^	% PR positive^a^
Virgin control	30.7 ± 1.35	31.6 ± 3.1

Group 1	26.5 ± 5.3	38.6 ± 8.5

Group 2	27.9 ± 7.5	28.4 ± 6.6

Group 3	29.3 ± 8.9	29.9 ± 3.0

Group 4	27.2 ± 3.6	14.6 ± 6.5*

One thousand cells were counted in mammary gland sections stained for 5BrdU and ER-α or 5BrdU and PR. ([^3^H]-thymidine and steroid receptors were counted for Group 4.) Group 1 tissue was collected at day 3 of pregnancy, Group 2 tissue was collected at day 4 of pregnancy, Group 3 tissue was collected at day 6 of pregnancy, and Group 4 tissue was collected at day 13 pregnancy after a second 5BrdU dose at day 4. Values are expressed as mean ± standard deviation. ^a^Double positive for 5BrdU and receptor/total 5BrdU positive. *P < 0.05 versus all groups individually. 5BrdU, 5-bromodeoxyuridine; ER, estrogen receptor; LREC, label-retaining epithelial cell; PR, progesterone receptor.

**Table 2:** Percentages of LREC within the receptor positive cell population

Time point	% ER-α-positive LREC population^a^	% ER-α-positive total population^a^	Ratio of % ER-α-positive LREC to total % ER-α-positive population^a^	% PR-positive LREC population^a^	% PR-positive total population^a^	Ratio of PR-positive LREC to total PR-positive population^a^
Non-pregnant	11.4 ± 3.2	16.1 ± 2.45	11.4/16.1 = 0.7081	14.1 ± 4.1	21.9 ± 5.96	14.1/21.9 = 0.6438

Group 1	10.1 ± 2.4	20.6 ± 5.92	10.1/20.6 = 0.4903	19.3 ± 5.9	22.4 ± 4.20	19.3/22.4 = 0.8616

Group 2	12.8 ± 7.9	16.1 ± 7.74	12.8/16.1 = 0.7950	18.0 ± 7.4	19.5 ± 4.23	18.0/19.5 = 0.9424

Group 3	13.7 ± 5.3	20.1 ± 5.64	13.7/20.1 = 0.6816	18.3 ± 10.1	14.3 ± 4.92^*†^	18.3/14.3 = 1.2797

Group 4	18.3 ± 8.1	6.62 ± 2.17*	18.3/6.62 = 2.7644	19.3 ± 9.4	6.5 ± 1.47^*†^	19.3/6.5 = 2.9692

One thousand cells were counted in mammary gland sections stained for 5BrdU and ER-α or 5BrdU and PR. ([^3^H]-thymidine and steroid receptors were counted for Group 4). Group 1 tissue was collected at day 3 of pregnancy, Group 2 tissue was collected at day 4 of pregnancy, Group 3 tissue was collected at day 6 of pregnancy, and Group 4 tissue was collected at day 13 of pregnancy after second 5BrdU dose at day 4. Values are expressed as mean ± standard deviation, or as %/% = ratio. ^a^Double positive for 5BrdU and receptor/total receptor positive. *P < 0.05 versus virgin control; ^†^*P *< 0.05 versus group 1. 5BrdU, 5-bromodeoxyuridine; ER, estrogen receptor; LREC, label-retaining epithelial cells; PR, progesterone receptor.

**Figure 7:**
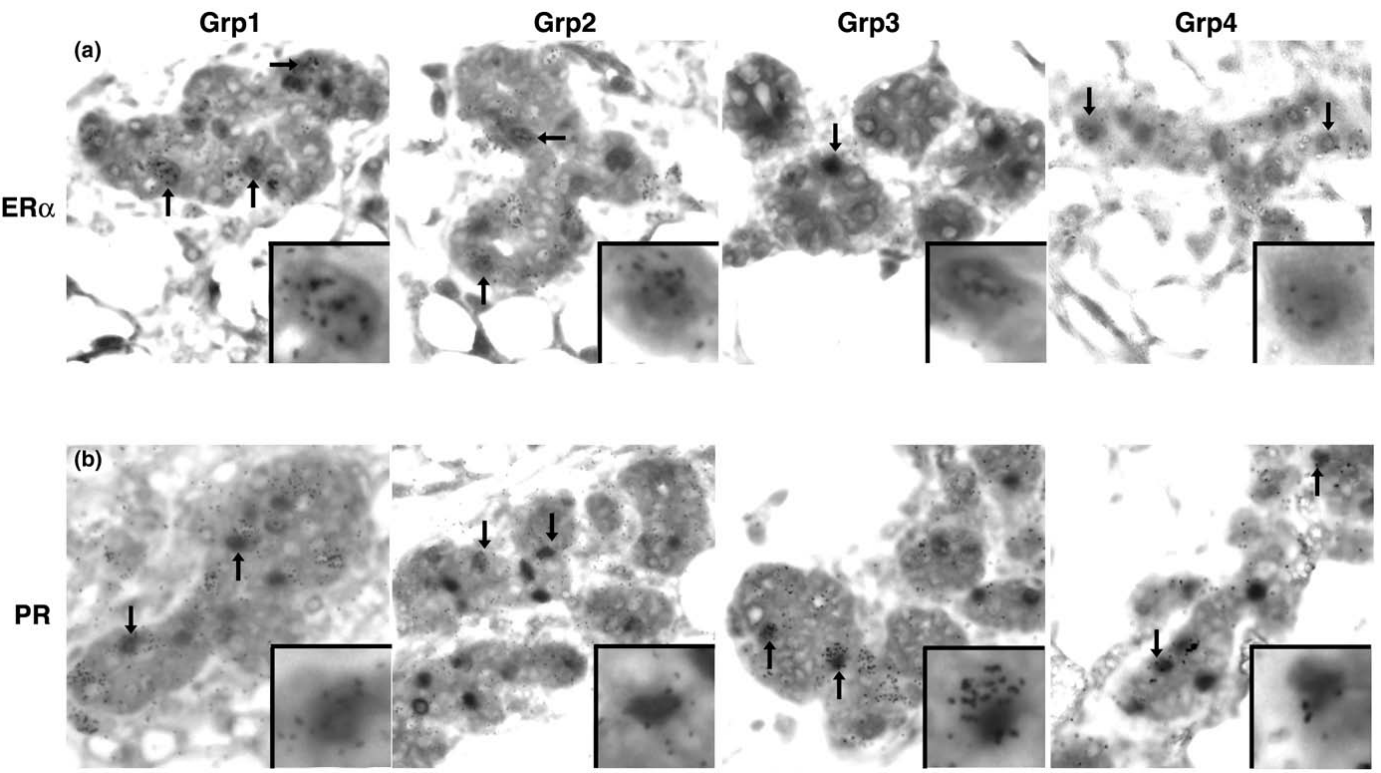
Nuclear steroid receptor expressing cells in cycle. The nuclear label [^3^H]-thymidine is detected by autoradiography in sections from all experimental groups that were also stained for the nuclear steroid receptors **(a) **ER-α or **(b) **PR. All experimental groups contained epithelial cells that were ER-α/[^3^H]-thymidine or PR/[^3^H]-thymidine double positive. A cell double positive for the nuclear steroid receptor and [^3^H]-thymidine within the tissue section are displayed in the inset. ER, estrogen receptor; PR, progesterone receptor.

An additional finding was that numerous ER-α-positive and PR-positive cells incorporated [^3^H]-thymidine, indicating that the nuclear steroid expressing cells are traversing the cell cycle. We found ER-α/[^3^H]-thymidine and PR/[^3^H]-thymidine double-positive cells in all four experimental groups (Figure 7). Because the [^3^H]-thymidine was given to the animals shortly before death in experimental groups 1 to 3, the only cells that would incorporate the nuclear label are those cells that are in cycle or just beginning to cycle through S-phase. The ER-α/[^3^H]-thymidine and PR/[^3^H]-thymidine double-positive cells found in group 4 (Figure 7, far right column) are different from those found in groups 1 to 3 because [^3^H]-thymidine defines the LRC and not 5BrdU. The [^3^H]-thymidine was given 8 days before the animals were killed, indicating that one of two scenarios occurred. In group 4, where 5BrdU defines cells in cycle, we found that 18% were 5BrdU positive, and of these more than one in five were doubly labeled with [^3^H]-thymidine. This demonstrates that a large proportion of the [^3^H]-thymidine retaining cell was traversing the cell cycle when 5BrdU was given. A small possibility is that the double-labeled cells observed on day 4 of pregnancy represent these double-labeled cells at day 13, but this is unlikely because the total alveolar population has increased approximately 8-fold to 10 fold [26].

## Discussion

The data presented in this study demonstrate that long label retaining cells within the murine mammary gland comprise numerous cell types, including undifferentiated stromal cells, luminal epithelial cells, myoepithelial cells, and neural and endothelial cells. We observed cycling LRC, as determined by incorporation of the second nuclear label, both inside (epithelial) and outside (nonepithelial) the basement membrane, and these cells persisted throughout early and mid-pregnancy. Additionally, we found that the expression patterns of ER-α and PR in LREC are altered in response to the hormonal changes during pregnancy.

These results are similar to our previous findings when we examined LREC after nulliparous animals had received injections of estrogen, progesterone, and prolactin for 5 days [21]. After treatments with estrogen, progesterone, and prolactin, we identified a significant increase in ER-α-positive LREC and a decreasing trend in LREC that are PR positive. We confirmed this observation in intact pregnant females in the present report. During pregnancy we observed a similar trend of increasing numbers of ER-α-positive cells and a significant decrease in PR-positive LREC in early pregnancy.

Stem cells that reside in the mammary gland are positioned in basal and suprabasal positions in the ducts and alveoli [15,27]. These mammary stem/progenitor cells are undifferentiated pale cells that are characterized by a lack of cytoplasmic organelles and their uncondensed chromatin. These cells have been characterized on the basis of their size into undifferentiated large light cells and small light cells (SLC). It is these cells that enter the cell cycle when cultured as mammary explants [28]. Additionally, these cells have been analyzed through the different stages of mammary development and the percentage of each morphotype calculated. These studies [15] revealed that the percentage of SLC in the general epithelial population did not change from puberty throughout pregnancy and into post-lactation involution, even though the number of mammary epithelial cells increased by 20-fold to 30-fold. This suggests that the numbers of SLC expand and contract coordinately with the total epithelial population, and implies that they have an essential role to play in the maintenance and expansion of the mammary epithelial cell population. The numbers of undifferentiated large light cells in the total population was much more variable, suggesting that they may represent a transitional state in the progression of SLC progeny to fully differentiated epithelial cells.

The hierarchy of stem/progenitor cells in mouse mammary tissue is comprised of three types of progenitor cells [18,28-30]: duct limited (no alveologenesis), lobule limited (no duct morphogenesis), and unlimited stem/progenitor cells. Mammary outgrowths that originate from either duct-limited or lobule-limited progenitors are comprised of myoepithelial and luminal epithelial cells, with a population of the luminal cells expressing ER and/or PR [30]. Additional experiments have demonstrated that entire mammary outgrowths are the progeny of a single cell that gives rise to all mammary cell types [26]. After multiple transplantation generations when growth senescence develops, the capacity to develop ducts or alveoli is lost independently [18]. Because each progenitor type gives rise to ER-positive and PR-positive cells, it is feasible that ER-positive and PR-positive epithelial cells arise from distinctly different antecedents during ductal growth and alveolar growth, because each probably perform different functions in mammary gland development and differentiation [31] (Smith GH, Medina D, unpublished observations). ER-α-null mice do not develop a ductal tree past the rudimentary tree present at birth, but when wild-type epithelium is transplanted into an ER-α-null fat pad it develops fully [32]. Our data demonstrate that populations of ER-α-positive and/or PR-positive cells are in cycle during pregnancy, whereas the rest of the nuclear steroid receptor expressing cells are not. ER-α-positive and/or PR-positive LREC may represent asymmetrically cycling LREC that are functionally distinct from ER-negative/PR-negative LREC. These observations suggest that cells among the ER-α-positive and PR-positive populations may arise from different antecedents and may be performing different functions within the gland.

Nuclear label retention for extended periods in individual cells may be explained by the presence of originally labeled cells that went out of cycle shortly after the label was administered. LRC may also represent cells that traverse the cell cycle very slowly. A recent study in which mammary epithelial cells were fluorescently sorted on the basis of proposed stem cell surface marker expression (CD24 and the α_6_β_1 _integrin complex) indicated that mouse mammary stem cells are ER-α, PR, and erbB2 negative [33], suggesting an undifferentiated phenotype. Undifferentiated cells are found in basal positions throughout the mammary gland [15], in a similar manner as for the undefined LRC described in this report.

Partially differentiated cells are observed in rapidly proliferating pregnant tissue, [^3^H]-thymidine labeled cells, and represent transit-amplifying cells committed to a secretory cell fate. We found a significant increase in the number of labeled ER-α-positive and an increasing number of labeled PR-positive cells (not statistically significant) in group 4 at day 12 of pregnancy, suggesting that the receptor-positive cells are in cycle or recently exited the cell cycle. Because no nuclear label had been added since day 4 of pregnancy, this indicates that some of the cells labeled at day 4 subsequently became ER-α-positive and/or PR-positive, and either stopped cycling or initiated asymmetric cellular division. Those cells that are double positive for 5BrdU and [^3^H]-thymidine represent the latter. It was recently hypothesized in a human model that nuclear steroid receptor positive cells exhibit stem cell characteristics of self-renewal by asymmetric division to produce transit-amplifying cells and differentiated progeny [34].

We previously reported the presence of periductal LRC that lie outside the basement membrane [6,21]. These cells do not cycle in mammary glands of mice treated with estrogen, estrogen/progesterone, or estrogen/progesterone/prolactin [6], but we found them in cycle during early pregnancy, which suggests that local signals may regulate their entry into the cell cycle. These cells do not express an endothelial marker (CD31), mesenchymal markers (SMA or desmin), or epithelial markers (cytokeratins, ER-α, or PR). They continue to cycle, as demonstrated by the uptake of [^3^H]-thymidine, at least 6 weeks after the final injection of 5BrdU. One possibility is that these LRC represent future ductal branching points or alveoli that form during pregnancy. Reversible adipocyte-to-epithelium and epithelium-to-adipocyte transdifferentiation has been demonstrated indirectly during pregnancy and involution [35]. It is known that there are three distinct types of progenitor cells in the murine mammary gland, and these periductal LRC may be examples of progenitor cells in a semidormant state, in which they are only involved in maintenance until the proper hormonal cues arise during pregnancy, at which time they initiate the formation of new ductal and lobule structure. This observation suggests that label-retaining progenitor cells might occupy specific microenvironmental locations.

Not only did we find numerous CD31-positive endothelial LRC lining the blood vessels in all of the groups (Figure 4), but we also found numerous endothelial LRC in pregnant mice that were cycling, as determined by [^3^H]-thymidine uptake (Figure 5a). This observation agrees with the increase in vasculature associated with pregnancy [36]. Proliferation and expansion of a tissue is stem/progenitor cell dependent, suggesting that the cycling endothelial LRC act as progenitor cells during pregnancy.

The implication of protection from mutation during DNA replication in cells that asymmetrically divide and reduced cancer risk was previously discussed [2]. So why then is breast cancer still one of the most prominent cancers in women? The continuous rounds of cellular proliferation and apoptosis during the menstrual cycle and any pregnancies create more possibilities for mutation during replication than most other tissues and organs, where cellular turnover is less frequent.

The present findings indicate that undefined cells, committed progenitor/transit-amplifying cells, and stem cells that take up nuclear label during allometric growth of the mammary gland and retain this label continue to cycle throughout pregnancy, and that ER-α and PR expression in these cells is affected by pregnancy.

## Conclusions

We conclude that long-lived cells characterized by their propensity to retain selectively their original DNA strands persist in adult mammary glands. This class of cells is represented in the mammary epithelium, in mammary-associated endothelium, and in neural tissue present in the fat pad. These cells are stimulated during pregnancy to enter the cell cycle and to contribute new progeny to their respective tissues through asymmetric divisions. During the expansion of the alveolar epithelium throughout pregnancy, new long label retaining epithelial cells, including ER-α-positive and PR-positive cells, arise among the expanding alveolar epithelium and continue to cycle asymmetrically while maintaining their originally labeled DNA copy. Our observations suggest that somatic cells destined to proliferate extensively in tissue homeostasis and, upon subsequent tissue expansion, undertake this role by adopting selective segregation of their original DNA in order to facilitate replacement or expansion of committed tissue cells that are devoid of genomic defects due to DNA replication.

## Abbreviations

5BrdU: 5-bromodeoxyuridine; ER: estrogen receptor; LRC: label-retaining cells; LREC: label-retaining epithelial cells; PR: progesterone receptor; SLC: small light cells; SMA: smooth muscle actin.

## Competing interests

The authors declare that they have no competing interests.

## Authors' contributions

GHS conceived of the study and its design and interpreted the data. BWB performed the collection of the data, conducted statistical analyses, interpreted the data, and wrote the manuscript. CAB performed the surgeries and interpreted the data. All authors read and approved the final manuscript.
